# A Systematic Review of Treatment Approaches for Individuals With Coexisting Diabetes and Substance Use Disorder

**DOI:** 10.7759/cureus.95352

**Published:** 2025-10-24

**Authors:** Bilal Husain, Suchismita Ray, Barbara Tafuto

**Affiliations:** 1 Department of Health Informatics, Rutgers University, Newark, USA

**Keywords:** buprenorphine-naloxone, care management, coexisting diseases, diabetes mellitus, glp-1 receptor agonists, semaglutide, substance use disorder, systematic review (syst. rev.), treatment approach

## Abstract

Diabetes is a chronic metabolic disease that is widespread and has negative consequences for health and well-being. Substance use disorder (SUD) is characterized by the uncontrolled use of substances, including tobacco, alcohol, marijuana, and illicit drugs, leading to impaired mental and physical health. Individuals with coexisting diabetes and SUD experience exacerbated consequences, including increased susceptibility to diabetes-related complications, hospitalizations, and mortality. Additionally, this unique population utilizes significant hospital resources, leading to a burden on the healthcare system. This systematic review is designed to synthesize current literature on individuals with coexisting diabetes and SUD, focusing on current treatments.

A systematic search of the PubMed and Embase databases was conducted using Medical Subject Headings and keyword strategies. After removing duplicates, studies were screened based on predefined inclusion and exclusion criteria, including relevance to the topic, publication within the past 10 years, availability of full-text articles, English language, and sufficient scope.

From an initial pool of 1,135 articles, 1,126 were excluded for not meeting eligibility, leaving nine retrospective studies for inclusion. Treatment strategies identified for individuals with coexisting diabetes and SUD encompassed pharmacotherapies (semaglutide and buprenorphine-naloxone), diligent adherence to SUD treatment, care management programs targeting individuals with multimorbidity, and reinforcers or rewards to either treatment adherence or drug abstinence. Although these approaches showed improvement in health outcomes, they primarily addressed diabetes and SUD separately. Additionally, few studies incorporated behavioral therapies, such as mindfulness-based interventions, despite their potential relevance to this population.

This review highlights the current treatment approaches for individuals with coexisting diabetes and SUD. The findings underscore the need for integrated care models that simultaneously address both conditions. Incorporating behavioral interventions alongside standard care strategies may offer a more comprehensive and effective treatment framework for this underserved population.

## Introduction and background

Burden of diabetes

Diabetes is a chronic and complex metabolic condition that affects both physical health and mental well-being [1]. It has become widespread and has reached significant proportions in the United States. In 2021, an estimated 38.4 million individuals (11.6% of the U.S. population) had diabetes, including 4.5% with undiagnosed cases [2]. Globally, 589 million adults are living with diabetes, a number projected to rise to 853 million by 2050 [3]. In terms of mortality, diabetes has contributed to the death of 3.4 million individuals in 2024 alone [3]. Diabetes is linked to decreased quality of life and frequently co-occurs with other chronic conditions such as cardiovascular disease and chronic kidney disease, which increases care complexity and healthcare costs [2,4]. Management of diabetes requires lifelong adherence to medication, insulin therapy, and dietary modifications; failure to maintain treatment often results in poor glycemic control and serious complications.

Burden of substance use disorders

Substance use disorder (SUD) is characterized by the compulsive use of substances such as alcohol, tobacco, opioids, and illicit drugs despite harmful consequences to health. A 2023 report by the National Institute on Drug Abuse noted that more than 40 million people in the United States were reported to have a SUD in 2020 alone, yet only 6.5% received treatment [5]. Substance use has been associated with psychiatric comorbidities, domestic violence, increased mortality, and a substantial burden on healthcare resources [6,7]. Cannabis use, in particular, has been known to cause earlier onset of psychotic conditions, while excess alcohol consumption contributes to hepatic, neurological disease, and several types of cancers [6]. Although pharmacological interventions exist to treat substance use, sustained abstinence remains difficult to achieve [8], and relapse following treatment can lead to adverse outcomes, including overdose deaths [9]. The burden of disease is magnified when SUD co-occurs with chronic diseases such as diabetes. For instance, while stimulants like cocaine are associated with cardiac and cerebrovascular disease, known diabetes complications, emerging evidence indicates that this risk is heightened in coexisting diabetes and SUD [6]. Additional details on the coexistence of diabetes and SUD, including prevalence and consequences, are discussed in subsequent sections.

Prevalence of coexisting diabetes and SUD

Research suggests that SUD is found to be more prevalent among individuals with diabetes mellitus [10,11] compared to those without diabetes [12]. Specifically, tobacco is frequently used by individuals with diabetes to reduce stress and improve cognitive abilities [1], while opioids are used to manage diabetic neuropathic pain [7]. Interestingly, alcohol, cannabis, and smoked tobacco are particularly common among younger adults with diabetes [12-14], whereas older adults tend to consume alcohol more than any other substance [15,16]. With the increasing number of U.S. states legalizing substances such as cannabis [13,14], the rising acceptance of illicit drug use [6], and the growing popularity of vaping as an alternative to cigarette smoking, there is heightened concern for individuals with diabetes who are already burdened by metabolic challenges. The widespread use of these substances within the diabetic population is worrisome, as it could lead to potential dependence, poorer disease management, and further decline in quality of life and overall health.

Consequences of coexisting diabetes and SUD

The intersection of diabetes and SUD creates a unique clinical population with even more complex treatment needs, higher healthcare resource utilization, and poorer health outcomes. Individuals with coexisting diabetes and SUD face a higher risk of diabetes-related complications [6,13,17-19], increased hospitalization [10,13], greater emergency department usage [20] and inpatient admissions [11], and mortality [6,10,13,17-19].

Beyond physical health, psychiatric conditions are more common, including mood, anxiety, personality, somatic, and schizophrenia [21,22]. Furthermore, individuals with both conditions are known to be unlikely to control HbA1c over time compared to those with diabetes alone [23], with alcohol use in particular linked to poorer adherence to diabetes medications [15,19]. Finally, individuals with both conditions use more healthcare resources and incur greater healthcare costs than those with either condition alone [1,11,13]. These patterns indicate that current treatment interventions may not effectively target both metabolic and behavioral needs, thereby lacking a holistic impact on health outcomes.

Gaps and objectives of this review

This review focuses on the coexistence of diabetes and SUD, a combination that presents unique clinical challenges. Existing literature indicates that individuals with diabetes may be at increased risk of substance use as a coping mechanism for chronic stress and neuropathic pain associated with diabetes [1,7]. Substance use in this context not only worsens existing metabolic conditions but also leads to compounded complications and poorer health outcomes. To our knowledge, while prior systematic reviews have examined treatment approaches for other chronic disease combinations, e.g., comorbid depression and diabetes [24], psychological disorder and somatic chronic disease [25], and depression and alcohol use [26], none have focused on treatment interventions tailored to coexisting diabetes and SUD, particularly in the past decade. Such a review can reveal both strengths and limitations of current treatment approaches, thereby guiding future research and clinical innovations to enhance care for this population. Therefore, this review aims to fill this gap by synthesizing existing treatment strategies for those with coexisting diabetes and SUD and identifying critical knowledge gaps to better address the needs of this underserved group.

## Review

Methods

Search Strategy

We searched the PubMed and Embase databases to identify peer-reviewed articles published in English within the past 10 years (January 2015 to August 2025). This timeframe was chosen to ensure the inclusion of the most up-to-date and relevant evidence on treatment approaches for individuals with coexisting diabetes and SUD. The final search was conducted in August 2025, prior to manuscript submission.

The databases were searched using a combination of Medical Subject Headings (MeSH) terms, Emtree terms, and various keywords. The search strategy included MeSH terms such as Diabetes Mellitus, Substance-Related Disorders, Emtree terms such as drug dependence, and keywords such as SUD, type 1 diabetes, type 2 diabetes, diabetes mellitus, Treatment*, intervention*, management, and therap*. Wildcard symbols (*) were applied to broaden the search, and boolean operators (AND, OR) were used to combine terms logically. Search syntax was customized based on the requirements for each database to ensure comprehensive retrieval of relevant articles. EndNote (Clarivate, London, UK) was used to identify and remove duplicates, while Microsoft Excel (Microsoft Corporation, Redmond, WA) was used to organize the articles and track the screening process. Below are the search equations that were used to retrieve articles from PubMed and Embase:

PubMed: ((Substance-Related Disorders[MeSH Terms]) OR ("substance use disorder")) AND ((Diabetes Mellitus[MeSH Terms]) OR ("type 1 diabetes") OR ("type 2 diabetes") OR ("diabetes mellitus")) AND ((Treatment*) OR (intervention*) OR (therap*) OR (therapy) OR (management))

Embase: ('drug dependence' OR 'substance use disorder') AND 'diabetes mellitus' AND ('therapy' OR 'disease management' OR 'intervention' OR 'drug therapy')

Inclusion Criteria

Articles were included if they were peer-reviewed, published in English between January 2015 and August 2025, and focused on individuals with coexisting diabetes (type 1 or type 2) and SUD with an emphasis on treatment, intervention, or management approaches.

Exclusion Criteria

Articles were excluded if they were more than 10 years old, not published in English, editorials, short surveys, book chapters, non-peer-reviewed documents, review articles, conference abstracts, non-human studies, lacked full text, or focused on diabetes or SUD without addressing both conditions.

Study Screening and Selection

The title and abstract screening was conducted by the primary author. Full-text articles were subsequently retrieved and reviewed to confirm inclusion. The screening and selection process followed Preferred Reporting Items for Systematic Reviews and Meta-Analyses (PRISMA) guidelines. It was documented step by step with a count of records identified, screened, excluded, and included in the final synthesis.

Data Extraction and Synthesis

The final list of articles was stored and organized using Microsoft Excel. Each study was assigned a unique identifier and coded for key study attributes, including author details, publication year, study design, sample size, and demographics (average age, gender distribution). Additional fields captured the type of diabetes (type 1 or type 2), type of SUD, details of treatment or intervention, and outcomes related to diabetes or SUD. Data extraction was conducted independently by the primary author, and findings were synthesized qualitatively to identify patterns, similarities, and gaps in treatment approaches.

Study Assessment and Risk of Bias

Risk of bias and methodological quality were assessed using the Newcastle-Ottawa Scale (NOS), which evaluates observational studies across three domains, namely selection of participants, comparability of study groups, and assessment of outcomes. Each retrospective study was independently reviewed and scored by the primary author across these domains. Total scores (0-9) were used to classify studies as low (7-9), moderate (5-6), or high (<5) risk of bias.

Statistical Analysis

Due to heterogeneity in study populations, interventions, and reported outcomes, a formal meta-analysis was not feasible. Instead, a qualitative narrative synthesis was conducted to describe and compare findings across studies. Although individual studies reported statistical effect measures such as odds ratios and hazard ratios, no new effect measures were calculated or pooled for this review. Likewise, statistical models (fixed or random effects), heterogeneity analysis, and publication bias assessments were not conducted, given the descriptive nature of this synthesis. This approach allowed for the structured comparison of treatment strategies across diverse studies, focusing on both diabetes-related and SUD-related outcomes.

Results

**Figure 1:**
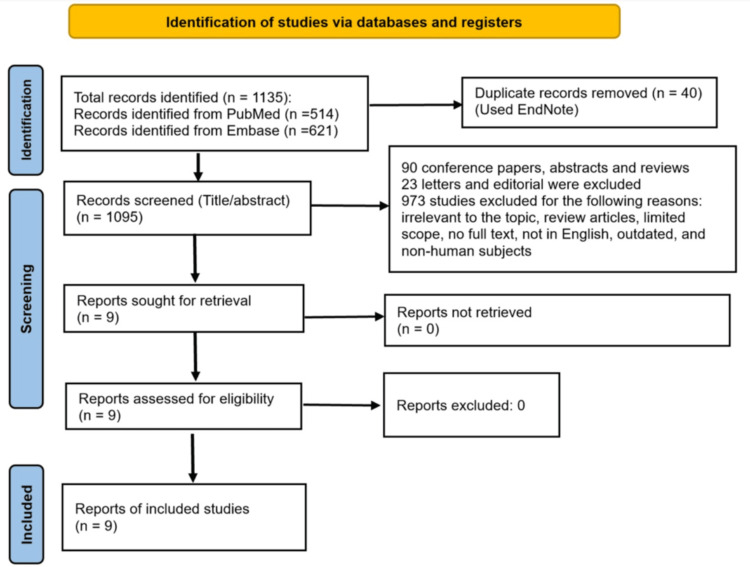
PRISMA diagram detailing the selection process to identify relevant studies PRISMA: Preferred Reporting Items for Systematic Reviews and Meta-Analyses

Out of the initial 1,135 studies retrieved, nine met the inclusion criteria. Out of the nine studies, eight were from the US, and one was from Canada. The articles were published between 2015 and 2025 and consisted of retrospective studies. The studies in this review included individuals with a diagnosis of type 1 or type 2 diabetes along with a coexisting SUD. Each of the studies assessed the effectiveness of different interventions or approaches in treating various diabetes-related or SUD-related health outcomes. Figure 1 was developed in accordance with the PRISMA guidelines.

Table 1 summarizes each included study, detailing the study design, year of publication, diabetes type, specific substances, and the geographic location where the study was conducted.

**Table 1:** Study summary of identified articles

Study	Study design	Substance of interest					Diabetes type			Location
		Tobacco	Alcohol	Cannabis	Opioids	Others	Type 1 diabetes	Type 2 diabetes	Unspecified	
Wang et al. [27]	Retrospective study	-	X	-	-	-	-	X	-	USA
Wang et al. [28]	Retrospective study	-	-	X	-	-	-	X	-	USA
Wang et al. [29]	Retrospective study	X	-	-	-	-	-	X	-	USA
Wang et al. [30]	Retrospective study	-	-	-	X	-	-	X	-	USA
Qeadan et al. [31]	Retrospective study	-	X	-	X	-	-	X	-	USA
Horigian et al. [23]	Retrospective study	X	X	-	-	X	-	X	-	USA
Tilbrook et al. [32]	Retrospective study	-	-	-	X	-	-	X	-	Canada
Forthal et al. [33]	Retrospective study	-	-	-	-	X	-	-	X	USA
Walter and Petry [34]	Retrospective study	-	X	-	X	X	-	-	X	USA

**Figure 2:**
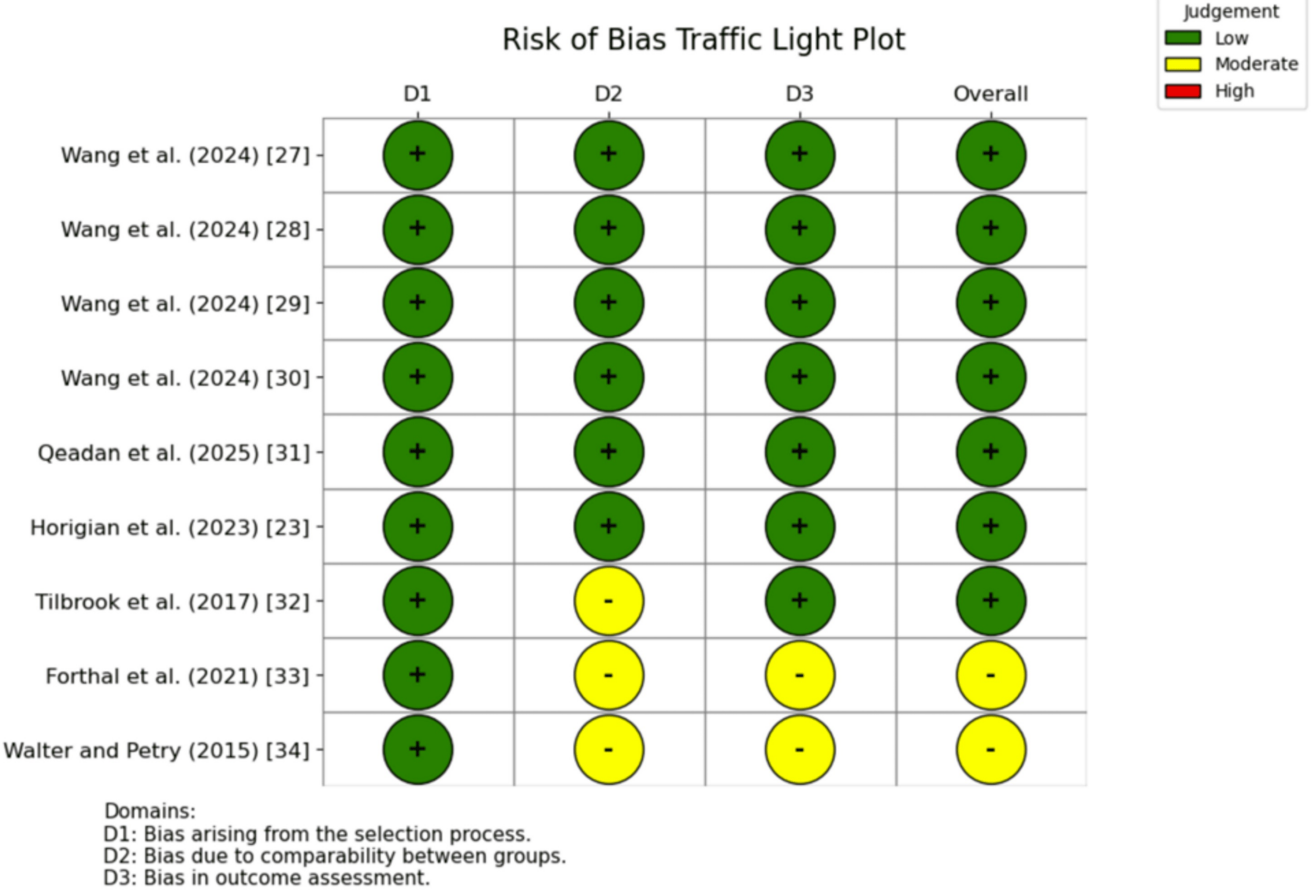
Traffic light plot showing risk of bias of included studies

For quality assessment, the NOS was used to evaluate the observational studies included in this review. Seven studies were rated as having an overall low risk of bias, while the remaining two were rated as moderate risk. Figure 2 illustrates a traffic light plot depicting risk of bias for the identified studies, and Table 2 presents a summary NOS assessment for each study.

**Table 2:** Summary of risk of bias of identified studies

Study	Selection (0-4)	Comparability (0-2)	Outcome (0-3)	Total score (0-9)	Risk of bias
Wang et al. [27]	4	2	3	9	Low
Wang et al. [28]	4	2	3	9	Low
Wang et al. [29]	4	2	3	9	Low
Wang et al. [30]	4	2	3	9	Low
Qeadan et al. [31]	4	2	3	9	Low
Horigian et al. [23]	4	2	3	9	Low
Tilbrook et al. [32]	4	1	3	8	Low
Forthal et al. [33]	3	1	2	6	Moderate
Walter and Petry [34]	3	1	2	6	Moderate

Demographic Characteristics

The mean age of study cohorts generally fell between the late 30s and mid‑70s, with semaglutide studies reporting older cohorts compared to other studies. Gender distribution also varied considerably, with female representation ranging from approximately 20%-68%. Women were least represented among alcohol using diabetes cohorts (approximately 20%-26%) and more prevalent in opioid use disorder (OUD) and type 2 diabetes groups (approximately 50%-68%). Overall, gender distribution varied widely by substance type and condition. Table 3 presents patient characteristics for each study, including the type of cohorts examined, sample size, mean age, and percentage of female participants for each cohort.

**Table 3:** Patient characteristics of the identified studies SUD: substance use disorder; OUD: opioid use disorder; AUD: alcohol use disorder; GLP-1 RA: glucagon-like-peptide-1 receptor agonists; NR: not reported ^*^The sample size, mean age, and female representation reported are individuals with diabetes who had a prior AUD diagnosis ^**^The sample size, mean age, and female representation reported are individuals with diabetes who had a prior CUD diagnosis ^***^Age and gender distribution are reported for the overall OUD and AUD cohorts; subgroup-specific demographics for participants with diabetes were not separately reported

Study	Cohort	Sample size (n)	Age (mean ± SD)	Female representation, n (%)
Wang et al. [27]	Semaglutide (alcohol users)	668^*^	57.1 ± 11.0^*^	175 (26.2)^*^
	Other antidiabetic medication (alcohol users)	21,445^*^	57.2 ± 11.5^*^	4,268 (19.9)^*^
Wang et al. [28]	Semaglutide (cannabis users)	254^**^	52.7 ± 11.3^**^	102 (40.2)^**^
	Non-GLP-1 RA antidiabetes medications (cannabis users)	7,942^**^	51.8 ± 12.2^**^	2,565 (32.3)^**^
Wang et al. [29]	Semaglutide (tobacco users)	5,967	58.5 ± 11.9	3,007 (50.4)
	Insulin (tobacco users)	213,225	64.6 ± 13.0	79,320 (37.2)
Wang et al. [30]	Semaglutide (opioids users)	3,034	57.4 ± 11.0	1,714 (56.5)
	Insulin (opioids users)	26,958	58.9 ± 12.3	11,889 (44.1)
Qeadan et al. [31]	Adults with OUD	503,747	50.5 ± 18.1^***^	257,415 (51.1)^***^
	Adults with AUD	817,309	46.8 ± 16.2^***^	263,991 (32.3)^***^
Horigian et al. [23]	Engaged in SUD treatment	6,108	NR	NR
	Not engaged in SUD treatment	770	NR	NR
Tilbrook et al. [32]	Type 2 diabetes and OUD on buprenorphine-naloxone	62	38.5 ± NR	42 (68.0)
	Type 2 diabetes only	511	51.8 ± NR	291 (57.0)
Forthal et al. [33]	Diabetes with SUD	7,733	50.0 ± 9.9	3,882 (50.2)
	Diabetes without SUD	12,066	52.0 ± 10.5	8,048 (66.7)
Walter and Petry [34]	SUD with diabetes	28	41.7 ± NR	9 (32.1)
	SUD without diabetes	653	39.5 ± NR	199 (30.5)

Current Treatment Approaches Related to Coexisting Diabetes and SUD

Multiple recent observational studies have demonstrated an association between glucagon-like peptide-1 receptor agonist (GLP-1 RA) use, particularly semaglutide, and reduced incidence and recurrence of SUDs among individuals with diabetes, regardless of prior SUD history. Wang et al. reported that, compared to other anti-diabetes medications, semaglutide was associated with a lower risk of incident alcohol use disorder (AUD) diagnosis among individuals with diabetes and no prior history of AUD diagnosis, and a lower risk of AUD recurrence among those with a prior AUD diagnosis [27]. Similarly, another 2024 study found that semaglutide was linked to reduced risk of incident cannabis use disorder (CUD) diagnosis among individuals with diabetes and no prior history of CUD diagnosis and reduced risk of CUD recurrence in those with a history of CUD [28]. For tobacco use disorder (TUD), semaglutide users were less likely to receive smoking cessation medication prescriptions and counseling, as well as fewer medical encounters related to a TUD diagnosis, compared to those using other antidiabetes medications [29]. Among individuals with coexisting diabetes and OUD, semaglutide was associated with a lower risk of opioid overdose compared to other antidiabetic medications [30]. Additionally, a retrospective analysis by Qeadan et al. found that, among individuals with type 2 diabetes, treatment with glucose-dependent insulinotropic polypeptide and/or GLP-1 RA was linked to reduced incidence of opioid overdose in those with OUD and fewer alcohol intoxications in those with AUD [31]. While these findings suggest a potential protective effect of GLP-1 RA, the retrospective design of these studies limits the ability to infer causality.

Beyond GLP-1 RA, several other strategies have been evaluated for those with coexisting diabetes and SUD. Two studies reported that participation in SUD treatment was linked to improved glycemic control in this population [23,32]. A retrospective study by Horigian et al. found that individuals with coexisting diabetes and SUD who participated in SUD or diabetic treatment were more likely to control HbA1c compared to those who did not participate in either form of care [23]. Similarly, among individuals with diabetes who used opioids, those who received buprenorphine-naloxone as opioid substitution therapy showed better glycemic control compared to those who did not receive the therapy [32]. Care management programs designed to prioritize high-risk patients were also shown to improve outcomes by enhancing access to essential services, with greater benefits observed in individuals with both diabetes and SUD compared to those with diabetes alone [33]. One study showed that contingency management, which provided reinforcers for either abstinence from drug use or adherence to treatment, resulted in improved health outcomes among individuals with coexisting diabetes and SUD, leading to longer periods of abstinence and a higher proportion of negative drug samples [34]. Taken together, these studies show that existing interventions can improve either diabetes-related outcomes, such as HbA1c control and glycemic management, or SUD-related outcomes, such as reduced overdose and substance use recurrence. However, none of the interventions targeted both conditions simultaneously, highlighting a gap in available treatment approaches for individuals with coexisting diabetes and SUD. Table 4 outlines the treatments evaluated in each study and summarizes their impact on health outcomes among individuals with coexisting diabetes and SUD.

**Table 4:** Treatments and outcomes from the identified studies SUD: substance use disorder; OUD: opioid use disorder; AUD: alcohol use disorder; CUD: cannabis use disorder; TUD: tobacco use disorder; HR: hazard ratio; OR: odds ratio; aOR: adjusted odds ratio; NYS-HH: New York State Health Homes; aIRR: adjusted incidence rate ratio; GIP: glucose-dependent insulinotropic polypeptide; GLP‐1 RA: glucagon-like peptide-1 receptor agonist; CM: contingency management

Study	Treatments	Groups	Outcomes	Metric	Results
Wang et al. [27]	Semaglutide	Individuals with type 2 diabetes mellitus with and without AUD	Incident and recurrent AUD diagnoses	HR	Incident AUD: HR = 0.56 (95% CI: 0.43-0.74). Recurrent AUD: HR = 0.61 (95% CI: 0.50-0.75)
Wang et al. [28]	Semaglutide	Individuals with type 2 diabetes mellitus with and without CUD	Incident and recurrent CUD	HR	Incident CUD: HR = 0.40 (95% CI: 0.29-0.56). Recurrent CUD: HR = 0.66 (95% CI: 0.42-1.03)
Wang et al. [29]	Semaglutide	Individuals with type 2 diabetes mellitus and TUD	Medical encounters for TUD diagnosis, smoking cessation medication prescription, and counseling	HR	Medical encounters for TUD diagnosis: HR = 0.68 (95% CI: 0.63-0.74). Smoking cessation medication prescription: HR = 0.32 (95% CI: 0.28-0.38). Smoking cessation counseling: HR = 0.69 (95% CI: 0.54-0.88)
Wang et al. [30]	Semaglutide	Individuals with type 2 diabetes mellitus and OUD	Opioid overdose risk	HR	Opioid overdose risk: HR = 0.32 (95% CI: 0.12-0.89)
Qeadan et al. [31]	GIP/GLP‐1 RA	Individuals with type 2 diabetes mellitus and OUD or AUD	Rates of opioid overdose and alcohol intoxication	aIRR	Rate of opioid overdose: aIRR = 0.62 (95% CI: 0.46-0.82). Rate of alcohol intoxication: aIRR = 0.51 (95% CI: 0.40-0.65)
Horigian et al. [23]	Diabetes or SUD treatment	Individuals with type 2 diabetes mellitus and SUD	HbA1c control (glycemic control)	OR	HbA1c control: among those engaged in diabetes treatment: OR = 1.85 (95% CI: 1.53-2.24). HbA1c control: among those engaged in SUD treatment: OR = 5.91 (95% CI: 5.05-6.91)
Tilbrook et al. [32]	Buprenorphine-naloxone	Individuals with type 2 diabetes mellitus and OUD	HbA1c control (glycemic control)	Percentage	Baseline HbA1c: 9.76%. HbA1c at two years with treatment: 8.57%
Forthal et al. [33]	Care management programs (NYS-HH)	Individuals with diabetes with and without SUD	Visits for eye exams, HbA1c tests, and medical attention for nephropathy, receipt of all three	aOR	Enrollment in NYS-HH was associated with greater improvements among those with SUD: eye exam (aOR = 1.08), HbA1c test (aOR = 1.20), nephropathy screening (aOR = 1.21), and all three metrics (aOR = 1.09)
Walter and Petry [34]	CM vs. standard care	Individuals with SUD with and without diabetes	Duration of abstinence, proportion of negative samples, and abstinence at nine-month follow-up	F-statistic	Patients with diabetes had longer abstinence durations and more negative samples under CM than nondiabetics, and were more likely to remain abstinent at nine months regardless of treatment. Duration of abstinence: F(1, 677) = 5.47, p < 0.05 and percent of negative samples: F(1, 677) = 5.52, p < 0.05

Discussion

Current Treatments and Lack of Integrated Approaches

Our review of treatment interventions for individuals with coexisting diabetes and SUD identified a spectrum of strategies, ranging from care management programs that prioritized patients with multimorbidity to reinforcement approaches promoting abstinence or treatment adherence and pharmacotherapies such as buprenorphine-naloxone and semaglutide. These treatment strategies were associated with improved health outcomes among individuals with coexisting diabetes and SUD, such as enhanced access to care, better glycemic control (HbA1c), and reductions in opioid overdose, alcohol intoxication, and incident or recurrent AUD, CUD, or TUD diagnosis. However, most interventions addressed each condition separately instead of together, indicating the need for integrated care. Prior work also illustrates this gap. For example, a review by López Zubizarreta et al. found that a combination of motivation, cognitive behavioral therapy, and pharmacotherapy could support smoking cessation among individuals with diabetes who smoke, without addressing whether these approaches also improve diabetes-related outcomes [35]. Similarly, in a semaglutide trial, researchers noted fewer smoking‑cessation prescriptions and counseling sessions, suggesting a reduced need for these interventions [29]. Yet, it remains unclear whether this decline reflected a true drop in tobacco use or a missed opportunity for integrated care, which warrants further investigation.

Although outside the scope of this review, related evidence suggests that certain therapies may influence both metabolic and substance-related outcomes. For example, a narrative review by Gillessen and Schmidt noted that silymarin, an extract from milk thistle seeds, improved liver function and reduced cirrhosis-related mortality among individuals with alcohol-related liver disease, while also improving glucose and HbA1c levels in patients with type 2 diabetes and coexisting liver cirrhosis [36]. While these effects were observed in separate populations, they highlight the potential for treatments like silymarin that simultaneously address metabolic and substance-related complications. Future research should evaluate whether agents such as silymarin and semaglutide can provide dual benefits in patients with coexisting diabetes and SUD, thereby helping to address the gap in integrated care.

Behavioral-Based Interventions

Finally, another notable gap in the literature is the absence of sufficient studies exploring the use of behavior-based therapies in improving outcomes among those with coexisting diabetes and SUD. Previous studies have shown that strategies like mindfulness-based interventions (MBI) can improve health outcomes for individuals with either diabetes or SUD. For instance, a study by Whitebird et al. showed that mindfulness-based stress reduction improved diabetes self-management, diabetes-related distress, glucose control, depression, anxiety, and stress among individuals with diabetes [37]. Similarly, a systematic review by Hamasaki found that MBIs improved glycemic control in individuals with diabetes [38]. Separately, an integrative review by Garland and Howard highlighted the significant impact of mindfulness in reducing craving and substance misuse, thereby supporting recovery from substance addiction [39]. Further, Ray et al. showed that a 10-minute guided mindfulness-oriented recovery enhancement meditation improved mood and had the potential to reduce acute stress- or cue-provoked drug craving [40]. Integrating such interventions into patient care for those with coexisting diabetes and SUD could improve outcomes across metabolic, psychiatric, and substance use domains. Future studies should take the opportunity to fill this gap by conducting randomized controlled trials to test the efficacy of MBIs in this unique population, as incorporating these approaches has the potential to reduce hospital burden while facilitating truly integrated care.

Strengths and Limitations

The studies in this review showed notable strengths. First, the studies used large, real-world data from national electronic health record networks, Medicaid programs, and community clinics, allowing them to include diverse and often underrepresented patient populations. Second, they applied strong research methods such as randomized trial designs, patient matching, and before-and-after comparisons to reduce bias and better estimate treatment effects. Third, they tracked outcomes over extended periods using reliable statistical tools like survival analysis and Cox regression, capturing both short and long-term impacts. Fourth, most studies conducted subgroup and sensitivity analyses to confirm that their findings were consistent across different patient groups and conditions. Finally, many studies shared their methods, code, and data definitions openly, improving transparency and enabling other researchers to build on their work.

However, several limitations were identified across the included studies. Many relied on data drawn from specific populations or health systems, limiting the generalizability of their findings. The predominance of retrospective observational designs also restricts the ability to infer causal relationships. Additionally, methodological concerns such as potential underdiagnosis or misdiagnosis of SUD, self-selection bias, missing data, and the presence of unmeasured or uncontrolled confounders may have influenced outcomes. Other notable limitations included short follow-up periods and the lack of data on key variables such as medication adherence, severity of substance use, craving, and withdrawal symptoms. Together, these limitations highlight the need for more robust, prospective studies including randomized controlled trials that use standardized methods, address missing clinical variables, and evaluate treatment outcomes through an integrated care lens for individuals with coexisting diabetes and SUD.

## Conclusions

Individuals with coexisting diabetes and SUD represent a distinct group facing heightened risks of diabetes and SUD-related complications, mortality, and other adverse health effects. Moreover, they place additional strain on healthcare systems due to increased demand for resources. With the growing prevalence of this demographic, it is essential that healthcare providers recognize and address their unique needs. This review highlighted a range of treatment strategies currently employed for this population. While many of these approaches led to improved outcomes, they often addressed diabetes and SUD separately, resulting in nonintegrated models of care. Furthermore, there was limited evidence on the use of behavioral therapies, such as MBIs, in treating individuals with both conditions. Incorporating behavioral approaches, either as standalone interventions or in combination with pharmacological and psychiatric treatments, may offer a more comprehensive and integrated strategy. Such approaches have the potential to simultaneously address the physical and psychological dimensions of coexisting diabetes and SUD, ultimately improving health outcomes and care delivery.
